# SLAB51 Probiotic Formulation Activates SIRT1 Pathway Promoting Antioxidant and Neuroprotective Effects in an AD Mouse Model

**DOI:** 10.1007/s12035-018-0973-4

**Published:** 2018-02-28

**Authors:** Laura Bonfili, Valentina Cecarini, Massimiliano Cuccioloni, Mauro Angeletti, Sara Berardi, Silvia Scarpona, Giacomo Rossi, Anna Maria Eleuteri

**Affiliations:** School of Biosciences and Veterinary Medicine, University of Camerino, Via Gentile III da Varano, 62032 Camerino, MC Italy

**Keywords:** Alzheimer’s disease, Oxidation, SIRT1, Probiotics

## Abstract

The gut-brain axis is a bidirectional communication network functionally linking the gut and the central nervous system (CNS). Based on this, the rational manipulation of intestinal microbiota represents a novel attractive therapeutic strategy for the treatment of CNS-associated disorders. In this study, we explored the properties of a probiotic formulation (namely SLAB51) in counteracting brain oxidative damages associated with Alzheimer’s disease (AD). Specifically, transgenic AD mice (3xTg-AD) were treated with SLAB51 and the effects on protein oxidation, neuronal antioxidant defence and repair systems were monitored, with the particular focus on the role of SIRT1-related pathways. We demonstrated that SLAB51 markedly reduced oxidative stress in AD mice brain by activating SIRT1-dependent mechanisms, thus representing a promising therapeutic adjuvant in AD treatment.

**Electronic supplementary material:**

The online version of this article (10.1007/s12035-018-0973-4) contains supplementary material, which is available to authorized users.

## Introduction

Alzheimer’s disease (AD) is a devastating form of dementia characterized by profound brain alterations and behavioural disorders. The histopathological hallmark of AD is the progressive accumulation of abnormal amyloid-β (Aβ) peptides and hyperphosphorylated tau protein in the brain of ageing subjects, as amyloid plaques and neurofibrillary tangles, respectively. In details, Aβ peptides are produced by proteolytic cleavage of the transmembrane amyloid precursor protein (APP), whereas tau is a brain-specific, axon-enriched protein normally associated with microtubules that loses its affinity for these structures upon phosphorylation [1]. Oxidative stress represents the mechanism through which Aβ neurotoxic peptides and tau protein mediate neurodegenerative processes and cause impaired synaptic plasticity, neuro-inflammation, neuronal and synaptic loss and neurotransmitter imbalance in AD [2] that ultimately correlates with the classical behavioural symptoms of AD [1].

Growing evidence supports the relevant role of oxidative stress in the onset and progression of AD: inadequate antioxidant defence systems, high O_2_ consumption, the presence of excitotoxic amino acids and high iron content promote the production of reactive oxygen and nitrogen species (ROS and RNS, respectively) in the brain [3, 4]. ROS and RNS are extremely unstable species that easily react with proteins, lipids, carbohydrates and nucleic acids, causing oxidative modifications that finally result in cellular dysfunctions [5, 6]. Comparative redox proteomic analysis of cerebrospinal fluid samples from mild cognitive impairment (MCI), the earliest clinical phase of AD, AD and control individuals identified specific oxidatively modified proteins early in the progression of AD [7]. Higher levels of protein carbonyls were detected in the hippocampus of 3xTg-AD mice as early as 3 months of age [8]. High levels of 4-hydroxy-2-nonenal (4-HNE), an extremely reactive aldehyde produced by membrane lipid peroxidation, were observed in the brain of MCI patients compared to controls [9, 10]. Interestingly, HNE was shown to bind both nicastrin, a component of the γ-secretase complex, and BACE, a β-secretase enzyme, differentially affecting their activity and thus altering APP processing [11]. Several authors reported on the occurrence of oxidative modifications to DNA (both nuclear and mitochondrial) [12] and RNA in the brain of AD subjects by detecting the levels of 8-hydroxy-2′-deoxyguanosine (8-oxodG), a marker of nucleic acid oxidation [13–15]. In addition, the increased oxidative profile has been often associated with a decrease both in the activity of antioxidant enzyme systems, including superoxide dismutase (SOD), catalase (CAT), glutathione peroxidase (GPx) and 8-oxoguanine DNA glycosylase-1 (OGG1) and in the levels of antioxidant molecules, among these glutathione [16–18].

Sirtuin-1 (SIRT1) is a NAD^+^-dependent protein deacetylase with established neuroprotective action, which is able to lower ROS levels and promote cell survival [19, 20]. In particular, SIRT1 protects cell from oxidative stress by modulating the transcription factors p53 and the forkhead O (FOXO) family [20–22]. Other mechanisms through which SIRT1 inhibits apoptosis include the deacetylation of Ku70, poly(ADP-ribose) polymerase-1 (PARP), Smad7 and HSF1 [19]. Interestingly, the activation of SIRT1-coupled retinoic acid receptor-β (RARβ) attenuated Aβ production in N2a cells expressing human APP Swedish mutation by increasing the activity of a disintegrin and metalloproteinase 10 (ADAM10)/α-secretase, suggesting a protective role for SIRT1 in AD [23].

In recent years, an increasing number of studies has been focusing on the role of the gut microbiota in disorders associated with the central nervous system (CNS), with special interest in the modulation of the gut-brain axis, a bidirectional system that integrates the gut and CNS activities [24, 25]. For this reason, the use of probiotics to modulate gut microbiota has been proposed for its positive effects in the prevention and treatment of allergies, gastrointestinal and urogenital tract infections, inflammatory disease, cystic fibrosis, certain type of cancers and, recently, also some CNS-associated disturbs [26]. Interestingly, the rational manipulation of intestinal microbiota in rats treated with VSL#3, a probiotic mixture containing eight bacterial strains, attenuated the age-related deficit in long-term potentiation and modulated the expression of several genes in the brain [27]. In a similar context, we recently demonstrated that 3xTg-AD mice administered with a novel formulation of lactic acid bacteria and bifidobacteria, namely SLAB51, exhibited evidence of attenuation of cognitive decline, reduction in Aβ aggregates and brain damages and partial restoration of impaired neuronal proteolytic pathways [28].

In this study, we further explored the role of SLAB51 in counteracting AD-associated symptoms with the specific aim of evaluating its ability to modulate brain oxidative status. For this purpose, 3xTg-AD mice were administered with SLAB51 and the effects on macromolecule oxidation, neuronal antioxidant defence and repair systems were assessed, with the focus on the role of SIRT1 pathways. Our data demonstrated that SLAB51 markedly reduces oxidative stress in AD mice brain by activating SIRT1-dependent mechanisms and therefore may be considered as a potential therapeutic adjuvant in AD treatment.

## Material and Methods

### Reagents and Chemicals

SLAB51 formulation was provided by Mendes S.A. (Lugano, Switzerland). PVDF membranes for Western blotting analyses were purchased from Millipore (Milano, Italy). Proteins immobilized on films were detected with the enhanced chemiluminescence (ECL) system (Amersham Pharmacia Biotech, Milano, Italy). SIRT1 (Merck Millipore, Darmstadt, Germany) and acetyl-p53(Lys379) antibodies were obtained from Cell Signaling Technology (Danvers, Massachusetts, USA). All other antibodies were from Santa Cruz Biotechnology (Heidelberg, Germany). The molecular probes’ protein molecular weight standards (6–205 kDa) (Sigma Aldrich, Italy) were used for molar mass calibration (it includes myosin 205 kDa, β-galactosidase 116 kDa, phosphorylase b 97 kDa, fructose-6-phosphate kinase 80 kDa, albumin 66 kDa, glutamic dehydrogenase 55 kDa, ovalbumin 45 kDa, carbonic anhydrase 30 kDa, trypsin inhibitor 21 kDa, lysozyme 14 kDa, aprotinin 6.5 kDa). Superoxide dismutase assay kit, reagents for antioxidant enzyme activities and protease inhibitors N-p-tosyl-phenylalanyl chloromethyl ketone (TPCK) and 4-(2-aminoethyl) benzenesulfonyl fluoride hydrochloride (AEBSF or Pefabloc) were from Sigma-Aldrich S.r.L. (Milano, Italy).

### Experimental Animals

The triple-transgenic mouse model of AD, B6;129-Psen1tm1Mpm Tg (APPSwe,tauP301L)1Lfa/J (namely, 3xTg-AD) and control wild-type animals were purchased from the Jackson Laboratory (Bar Harbor, Maine, USA). 3xTg-AD mice were previously characterized and represent a reliable model of human AD patients. In this model, Aβ intracellular immunoreactivity can be detected in some brain regions as early as 3 to 4 months of age [29]. Experiments were conducted using 8-week-old male mice (weight 15–25 g) in accordance with the guidelines of the European Communities Council (86/609/ECC) for the care and use of laboratory animals. Mice were housed in plastic (Makrolon) cages (four animals per cage) in a temperature-controlled room (21 ± 5 °C) and 60% humidity on 12 h light/dark inverted cycle (light was switched on at 8:00 P.M.) and maintained on laboratory diet (Mucedula, Italy) with water ad libitum. All appropriate measures were taken to minimize pain and discomfort in experimental animals.

### SLAB51 Administration

Eight-week-old male 3xTg-AD mice (*n* = 64) were divided in two groups: one was administered for 16 weeks with SLAB51 in water, and the control group was administered with water. Simultaneously, 64 age-matched wild-type (wt) mice were divided into wt control and wt-treated groups. SLAB51 is a formulation made of nine live bacterial strains (*Streptococcus thermophilus*, bifidobacteria *(Bifidobacterium longum*, *B. breve*, *B. infantis*), lactobacilli (*Lactobacillus acidophilus*, *L*. *plantarum*, *L. paracasei*, *L. delbrueckii* subsp. *bulgaricus*, *L. brevis*)). The dosage (200bn bacteria/Kg/day) was determined using the body surface area normalization [30]. The body weight was measured every 2 weeks before starting the treatment, then several times a week during the treatment to ensure single-housed animals received the proper intake of the experimental food. Preliminary studies were performed to evaluate both viability and stability of the probiotic formulation upon solubilisation in water at 21 ± 5 °C. The percentage of vital bacteria was determined by fluorescence microscopy, which revealed that 88% of the strains survived after 30 h under the abovementioned conditions. Thus, the drinking solution was changed every day replacing the bottles with fresh solutions. Mice were sacrificed for biochemical analyses at 12, 18 and 24 weeks of age, and the brains were properly stored at − 80 °C.

### Preparation of Brain Extracts

Mouse brain tissues were homogenized in 50 mM Tris buffer, 150 mM KCl, 2 mM EDTA, pH 7.5 (1:5 weight:volume of buffer). Homogenates were immediately centrifuged at 13,000×*g* for 20 min at 4 °C, and supernatant and pellet fractions were collected. Protein content was determined by the Bradford method [31] using bovine serum albumin (BSA) as standard.

### Sirtuin-1 Activity

SIRT1 activity was determined using the fluorescent SIRT1 substrate (Lys379/382 residues of p53), Arg-His-Lys-Lys-(ɛ-acetyl)-AMC (Cayman, Vinci-Biochem, Italy). The assay was performed by incubating the substrate at a final concentration of 125 μM, brain homogenates (30 μg of total protein content) and 3 mM NAD^+^ in the assay buffer (50 mM Tris-HCl, pH 8.0, containing 137 mM sodium chloride, 2.7 mM KCl and 1 mM MgCl_2_). Upon 1 h of incubation at 37 °C, the deacetylated product was cleaved by trypsin [32] and the fluorescence of the released AMC was recorded on a Spectramax Gemini XPS microplate reader (*λ*_exc_ 365 nm, *λ*_em_ 449 nm).

### Analysis of RARβ Acetylation

RARβ was immunoprecipitated from brain homogenates, and acetylated lysines were immunodetected. In detail, aliquots of brain homogenates were incubated with agarose-conjugated protein A immobilized on Sepharose CL-4B (Sigma-Aldrich S.r.L., Milano, Italy), followed by SDS-PAGE and immunoblotting. After reconstituting the agarose-conjugated protein A in deionized water, it was washed in washing buffer (Tris 50 mM, NaCl 150 mM, BSA 0.1%, pH 8) and centrifuged at 12,000×*g* for 10 s at room temperature. The supernatant was discarded and the agarose conjugate was re-suspended in washing buffer and finally aliquoted in several 100-μL fractions that were added with 2 μg of the primary antibody; the tubes were incubated in a shaker for 60 min at room temperature and then centrifuged at 3000×*g* for 2 min at 4 °C. Again, supernatants were discarded, washing steps with 1 mL of washing buffer were performed and 500 μg of total protein brain homogenates were added to each tube. After overnight incubation in a rotating mixer at 4 °C, centrifugation, discarding and washing steps were performed, to obtain the pellets to be analysed by SDS-PAGE. Pellets were suspended in 40 μL Laemmli sample buffer, heated at 95 °C for 5 min and then centrifuged at 12,000×*g* for 30 s at room temperature. Finally, 15 μL of the collected supernatant, containing immunoprecipitated RARβ, were analysed by Western blotting.

### Redox Enzyme Activity Assays

*Catalase* (CAT) activity was determined in brain homogenates following the Aebi protocol with minor modifications [33]. Briefly, 20 μL of homogenate was added to 50 mM potassium phosphate buffer (pH 7.0) and 9 mM H_2_O_2_. The decrease in absorbance at 240 nm was monitored during the first minute of incubation at 25 °C on a Varian Cary 1E spectrophotometer, and the Cary Win UV software was used to analyse kinetic raw data. CAT activity was expressed as μmol/min/mL of protein.

*Superoxide dismutase* (SOD) catalyses the dismutation of the superoxide anion (${\mathrm{O}}_2^{\cdot -}$) to H_2_O_2_ and O_2_. SOD activity was measured in brains using the SOD Assay Kit-WST (Sigma-Aldrich) following the manufacturer’s protocol, and the change in absorbance at 440 nm was read on a Varian Cary 1E spectrophotometer.

*Glutathione S-tranferase* (GST) assay is based on the monitoring of the increase in absorbance at 340 nm after conjugation of the thiol group of GSH to the 1-chloro-2,4-dinitrobenzene (CDNB) substrate [34–36]. Briefly, 5 μL of brain homogenate were added to a mixture containing 0.2 M reduced GSH, 0.2 M CDNB (previously dissolved in DMSO) and potassium phosphate buffer (0.3 M, pH 6.5). The absorbance at 340 nm was monitored for 4 min (*t* = 25 °C). GST activity was expressed as μmol/min/mL of protein.

*Glutathione peroxidase* (GPx) activity assay is based on the reduction of tert-butyl hydroperoxide (TBH) under GSH oxidation and subsequent reduction of GSSG by glutathione reductase in the presence of NADPH. Briefly, 50 μL of the homogenized sample were added to the mixture containing 50 mM Tris-HCl, pH 8.0, 0.5 mM EDTA, 5 mM NADPH and 42 mM reduced GSH. The decrease in NADPH absorbance measured at 340 nm during its oxidation to NADP^+^ is related to GPx activity, since GPx is the rate limiting factor of the coupled reactions [37, 38]. Reaction was monitored at 25 °C for 4 min. After 1 min of incubation, 30 mM H_2_O_2_ was added to start the reaction. The activity is expressed as units of enzyme that catalyse the oxidation of one μmole of NADPH per min.

### Western Blotting Analyses

The levels of 3-nitrotyrosine (3-NT), 4-HNE, 8-oxodG, OGG1, PARP1, p53 and acetyl-p53, catalase, SOD, GST and GPx in brain homogenates were analysed by Western blotting assays. In detail, for each time point, brain homogenates (20 μg total protein) were subjected to SDS-PAGE on 12% gels (10% for LC3) and electroblotted onto PVDF membranes. Successively, upon incubation with specific antibodies, the immunoblot detections were carried out with ECL Western blotting analysis system. Molecular weight markers (6.5–205 kDa) were included in each gel. Glyceraldehyde-3-phosphate dehydrogenase (GAPDH) was used to check equal protein loading.

### Oxyblot Analysis

Protein carbonyl groups were determined with the Oxyblot kit (Appligene-Oncor, Strasbourg, France). Brain homogenates (15 μg of total proteins) were incubated at room temperature with 2,4-dinitrophenylhydrazine (DNPH) to form 2,4-dinitrophenylhydrazone (DNP-hydrazone), according to the manufacturer data sheet. Then, the DNPH-derivatized samples were separated by SDS-PAGE and electroblotted onto PVDF membranes. Transferred membranes were sequentially incubated with an anti-DNP antibody and a secondary specific antibody. The detection was performed with the ECL system [39]. In order to check equal protein loading, reversible Ponceau staining was used prior to incubation with the anti-DNP primary antibody. Statistical significance was calculated considering the ratio between densitometric values of the oxyblot bands (oxidation level) and those stained with Ponceau red (protein content).

### Statistical Analysis

Statistical analysis was performed with one-way ANOVA, followed by the Bonferroni post hoc test using Sigma-stat 3.1 software (SPSS, Chicago, IL, USA). *P* values < 0.05 were considered to be significant.

## Results

### SLAB51 Activates SIRT1 Pathway in AD Mice

**Fig. 1:**
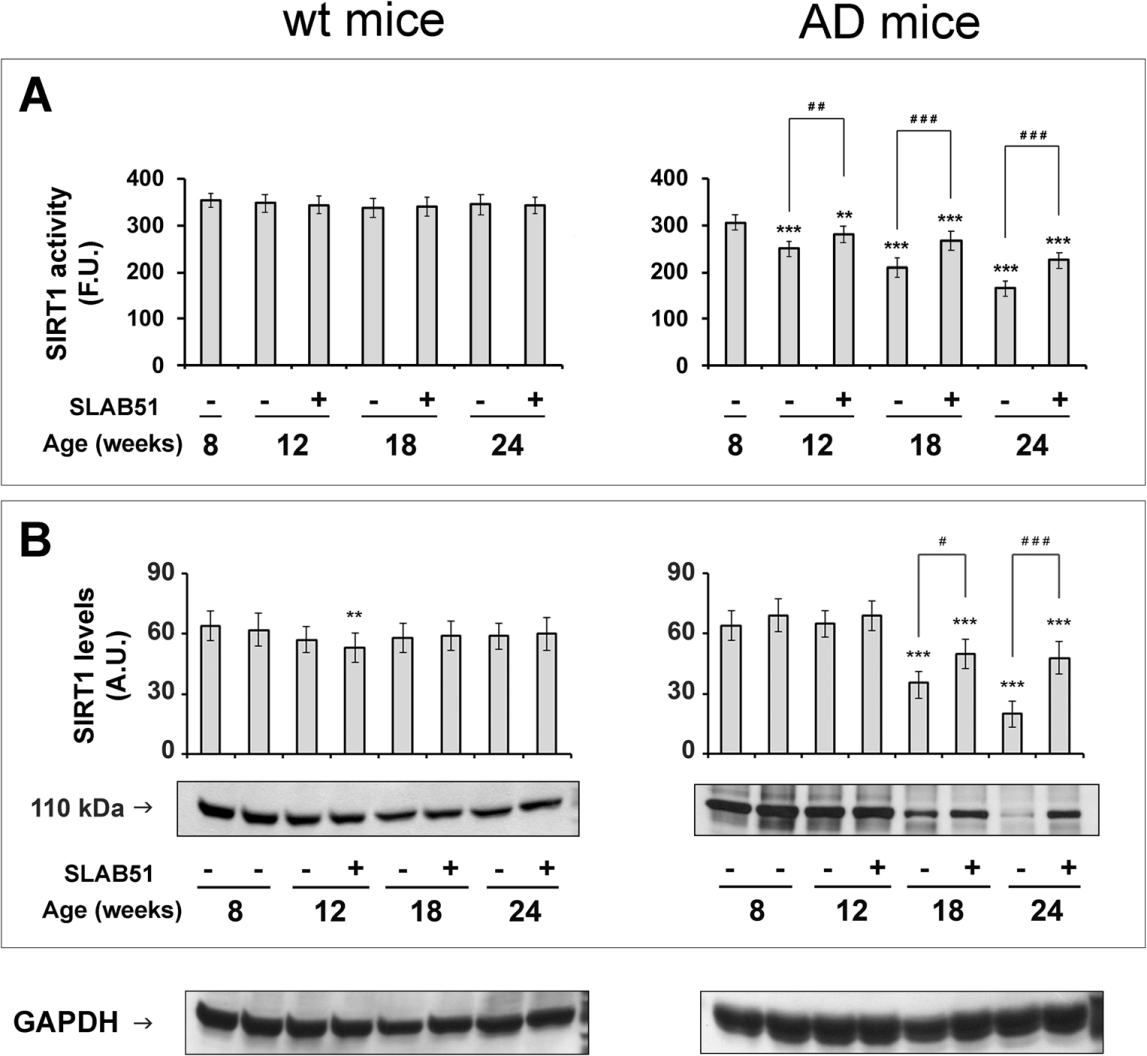
Effect of SLAB51 on SIRT1 activity and expression. SIRT1 activity (**a**) and expression levels (**b**) measured in brain homogenates of SLAB51-treated and SLAB51-untreated wt (left) and AD (right) mice. The enzyme activity is expressed as fluorescent units (F.U.). The densitometric analyses obtained from five separate blots and representative immunoblots are shown. Equal protein loading was verified by using an anti-GAPDH antibody. The detection was performed with an ECL Western blotting analysis system. Statistical significance compared to untreated 8-week-old mice and age-matched mice is indicated with asterisks (*p < 0.05; **p < 0.01; ***p < 0.001) and hashtags (^#^*p* < 0.05; ^##^*p* < 0.01; ^###^*p* < 0.001), respectively

We first explored the effects of SLAB51 on the SIRT1 pathway. SIRT1 activity and expression remained unchanged in wt mice throughout the experimentation, regardless of the treatment. The only minor, still significant decrease was observed only in SLAB51-treated wt mice at 18 weeks of age (Fig. 1, left plots).

SIRT1 profile was markedly modified in transgenic mice brains: specifically, SIRT1 activity progressively decreased with animal ageing, and SLAB51 treatment partially re-established enzyme functionality (Fig. 1a). This evidence was in line with the parallel changes in SIRT1 expression (SLAB51 treatment significantly counteracted the dramatic decrease in SIRT1 levels in 18- and 24 week-old AD mice) (Fig. 1b). Conversely, SIRT1 activity did not change at any time point in both control and age-matched treated wt mice (Fig. 1a, left box).

**Fig. 2:**
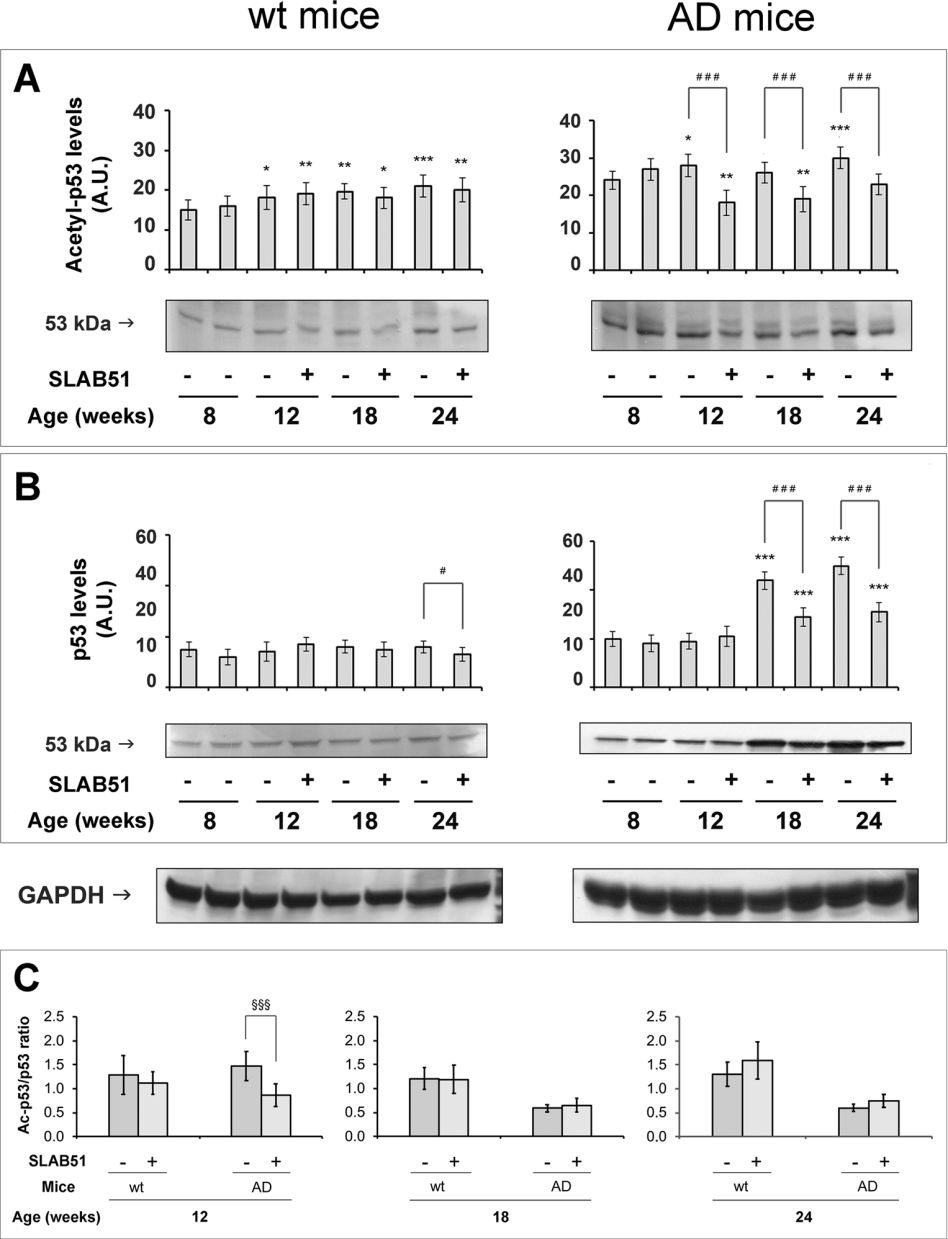
Effect of SLAB51 on p53. Acetylated p53 (**a**) and p53 (**b**) levels measured in brain homogenates of SLAB51-treated and SLAB51-untreated wt (left) and AD (right) mice. The densitometric analyses obtained from five separate blots and representative immunoblots are shown. Equal protein loading was verified by using an anti-GAPDH antibody. The detection was performed with an ECL Western blotting analysis system. Statistical significance compared to untreated 8-week-old mice and age-matched mice is indicated with asterisks (*p < 0.05; **p < 0.01; ***p < 0.001) and hashtags (^#^*p* < 0.05; ^##^*p* < 0.01; ^###^*p* < 0.001), respectively. (**c**) Pairwise comparison of the effects of SLAB51 in Ac-p53/p53 ratios in wt and AD ageing mice. Statistical significance compared to age-matched mice is indicated with “^§^” mark (^§^*p* < 0.05; ^§§^*p* < 0.01; ^§§§^*p* < 0.001)

**Fig. 3:**
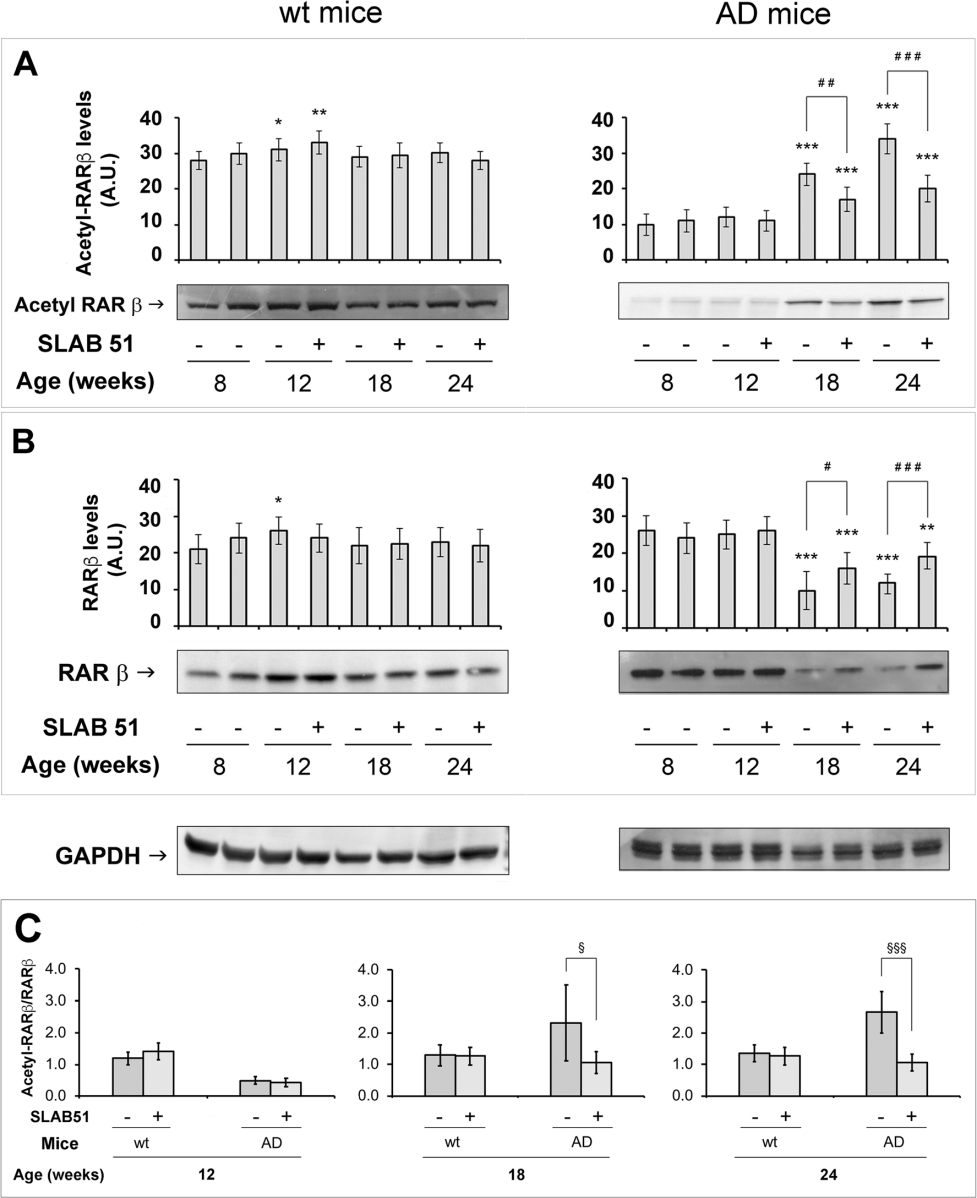
Effect of SLAB51 on RARβ. Acetylated RARβ (**a**) and RARβ (**b**) levels measured in brain homogenates of SLAB51-treated and SLAB51-untreated wt (left) and AD (right) mice. The densitometric analyses obtained from five separate blots and representative immunoblots are shown. Equal protein loading was verified by using an anti-GAPDH antibody. The detection was performed with an ECL Western blotting analysis system. Statistical significance compared to untreated 8-week-old mice and age-matched mice is indicated with asterisks (*p < 0.05; **p < 0.01; ***p < 0.001) and hashtags (^#^*p* < 0.05; ^##^*p* < 0.01; ^###^*p* < 0.001), respectively. **c**: Pairwise comparison of the effects of SLAB51 in Ac-RARβ/RARβ in wt and AD ageing mice. Statistical significance compared to age-matched mice is indicated with “^§^” mark (^§^*p* < 0.05; ^§§^*p* < 0.01; ^§§§^*p* < 0.001)

The recovery of SIRT1 functionality was also evaluated by monitoring the levels of two protein substrates, namely RARβ and p53, both in the acetylated and non-acetylated forms (Fig. 2 and Fig. 3). p53 is one of the targets of SIRT1 and, upon deacetylation, it is negatively controlled repressing the p53-dependent apoptosis [40], whereas RARβ promotes the non-amyloidogenic pathway for APP processing, and its activation due to deacetylation favours the transcription of ADAM10, which encodes for α-secretase [23]. In AD mice, SLAB51 promoted the decrease in acetylated-p53 levels already detectable in 12-week-old animals (Fig. 2, right boxes). Additionally, probiotic administration exerted an interesting effect on total p53, whose levels decreased in 18- and 24-week-old AD mice. Control and age-matched treated wt mice did not show any variations in both p53 and acetyl-p53 levels (Fig. 2, left boxes). Acetylated RARβ levels increased time-dependently and RARβ decreased in untreated AD mice at 18 and 24 weeks of age compared to control (Fig. 3, right panels).

In good agreement with data on SIRT1 activity/expression, the progressive increase in Ac-RARβ level and the concomitant decrease in RARβ in untreated AD mice of 18 and 24 weeks were significantly counteracted by SLAB51 treatment, which restored the 8-week-old AD mice ratio between acetylated and non-acetylated forms (Fig. 2c). SIRT1 differently affected the balance between Ac-p53 and p53, with significant beneficial effects being observed only at the earliest stage of treatment (Fig. 3c).

### Effect of SLAB51 Mixture on Antioxidant Enzymes

**Fig. 4:**
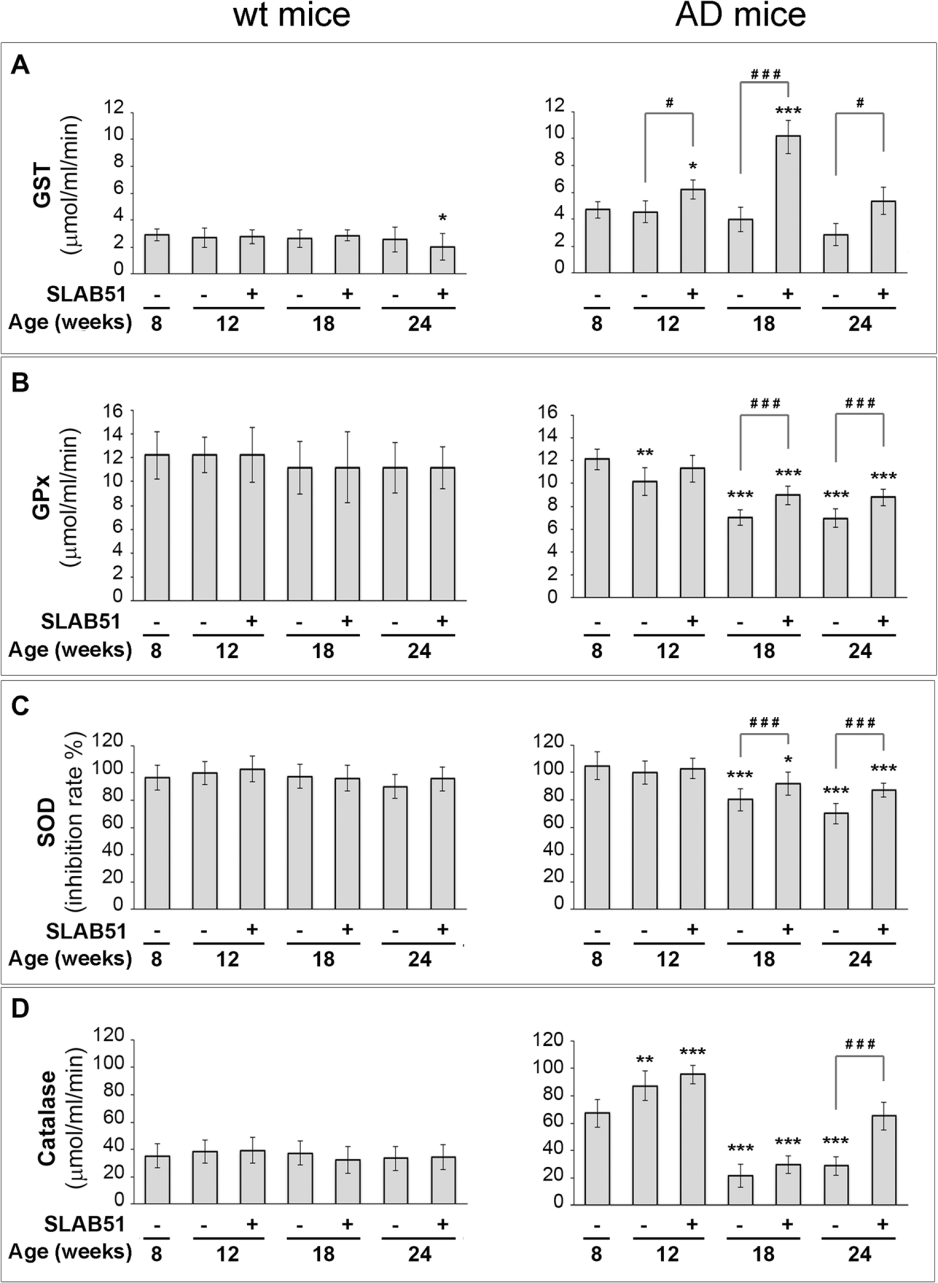
Effect of SLAB51 on the activity of antioxidant enzymes. GST (**a**), GPx (**b**), SOD (**c**) and CAT (**d**) activities measured in brain homogenates of SLAB51-treated and SLAB51-untreated wt (left) and AD (right) mice (see “Materials and Methods” section for further details). Results are expressed as fluorescence units (F.U.). Statistical significance compared to untreated 8-week-old mice and age-matched mice is indicated with asterisks (*p < 0.05; **p < 0.01; ***p < 0.001) and hashtags (^#^*p* < 0.05; ^##^*p* < 0.01; ^###^*p* < 0.001), respectively

GST, GPx, SOD and CAT activities were measured in brain homogenates from both wt and transgenic animals. Redox enzyme activities were constant at all time points in wt mice, regardless of the treatment with SLAB51 (Fig. 4, left insets). Only a minor, still significant decrease was observed at 24 weeks of age in GST activity of treated wt mice (Supp. Fig. 2, right panel). The treatment with SLAB51 significantly modified the activity profile of these redox enzymes in AD mice.

Generally, SLAB51 significantly affected the activity of redox enzymes in AD mice at intermediate-to-late time points (Supp. Fig. 3). More specifically, in untreated AD mice, GST activity did not significantly change in ageing animals, and SLAB51 treatment induced a substantial increase with time at 12 and 18 weeks of age. No significant change was induced in 24-week-old AD mice (Fig. 4a).

SLAB51 treatment counteracted the observed age-dependent decrease in GPx activity with ageing at all time points (Fig. 4b). Interestingly, the impaired enzymatic functionalities were associated with a time-dependent increased expression of these enzymes in AD subjects (data not shown).

In untreated AD mice, SOD activity significantly decreased in ageing animals more evidently at intermediate-to-late time points, and SLAB51 treatment partially helped recovery basal levels of 8-week-old untreated AD mice. (Fig. 4c). Again, catalase activity decreased with time in untreated AD mice starting from 18 weeks of age, and only long-term treatment with SLAB51 was effective in restoring basal activity of 8-week-old untreated AD mice (Fig. 4d).

Interestingly, the effects observed on SOD and GPx activities in untreated AD animals were in line with the significantly compromised activity/expression of SIRT1 at 18 and 24 weeks of age. Analogously, the observed recovery in the activity of these redox enzymes upon probiotic administration could be compatible with the partially restored activity/expression of SIRT1 (Supplementary Figs. 4 and 5).

Globally, the increased activity of these enzymes upon SLAB51 administration demonstrated the beneficial antioxidant effects exerted by the probiotic formulation in AD mice brains.

### Effect of SLAB51 Mixture on Protein and Lipid Oxidation

**Fig. 5:**
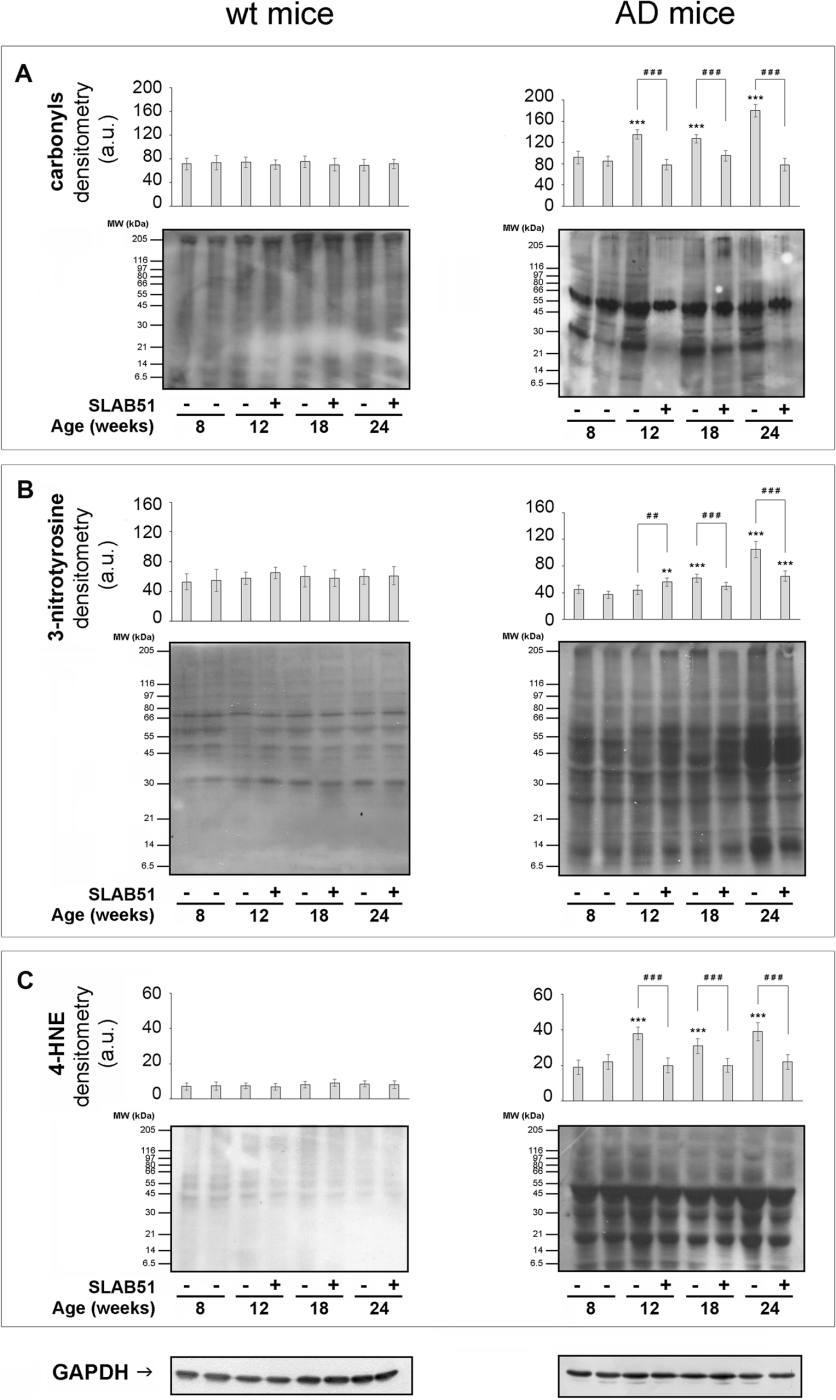
Effect of SLAB51 on protein and lipid oxidation. Protein carbonyls (**a**), 3-NT (**b**) and 4-HNE adduct (**c**) levels measured in brain homogenates of SLAB51-treated and SLAB51-untreated wt (left) and AD (right) mice. The densitometric analyses obtained from five separate blots and representative immunoblots are shown. Equal protein loading for 3-NT (**b**) and 4-HNE adducts (**c**) was verified by using an anti-GAPDH antibody. Ponceau staining has been used to check loading in oxyblot, as reported in the “Materials and Methods” section (**a**, staining not shown). The detection was performed with an ECL Western blotting analysis system. Molecular weight standards (6–205 kDa) were used for molar mass calibration (myosin 205 kDa, β-galactosidase 116 kDa, phosphorylase b 97 kDa, fructose-6-phosphate kinase 80 kDa, albumin 66 kDa, glutamic dehydrogenase 55 kDa, ovalbumin 45 kDa, carbonic anhydrase 30 kDa, trypsin inhibitor 21 kDa, lysozyme 14 kDa, aprotinin 6.5 kDa). Statistical significance compared to untreated 8-week-old mice and age-matched mice is indicated with asterisks (*p < 0.05; **p < 0.01; ***p < 0.001) and hashtags (^#^*p* < 0.05; ^##^*p* < 0.01; ^###^*p* < 0.001), respectively

The effect of SLAB51 on the levels of carbonyl groups, 3-NT and HNE-adducts (all established markers of protein and lipid oxidation), confirmed the strong antioxidant potential of the probiotic formulation. No change with time or treatment was observed in the levels of protein/lipid oxidation markers here considered in wt mice (Fig. 5, left insets). Generally, carbonyls, 3-NT and 4-HNE levels increased with ageing in untreated AD mice. SLAB51 administration effectively restored basal levels of carbonyls and 4-HNE at all time points (Fig. 5a, c), whereas it was less effective on 3-NT, which was significantly decreased only at 18 and (partially) at 24 weeks of age (Fig. 5b).

### Effect of SLAB51 Mixture on DNA Oxidation and DNA Repair Mechanisms

**Fig. 6:**
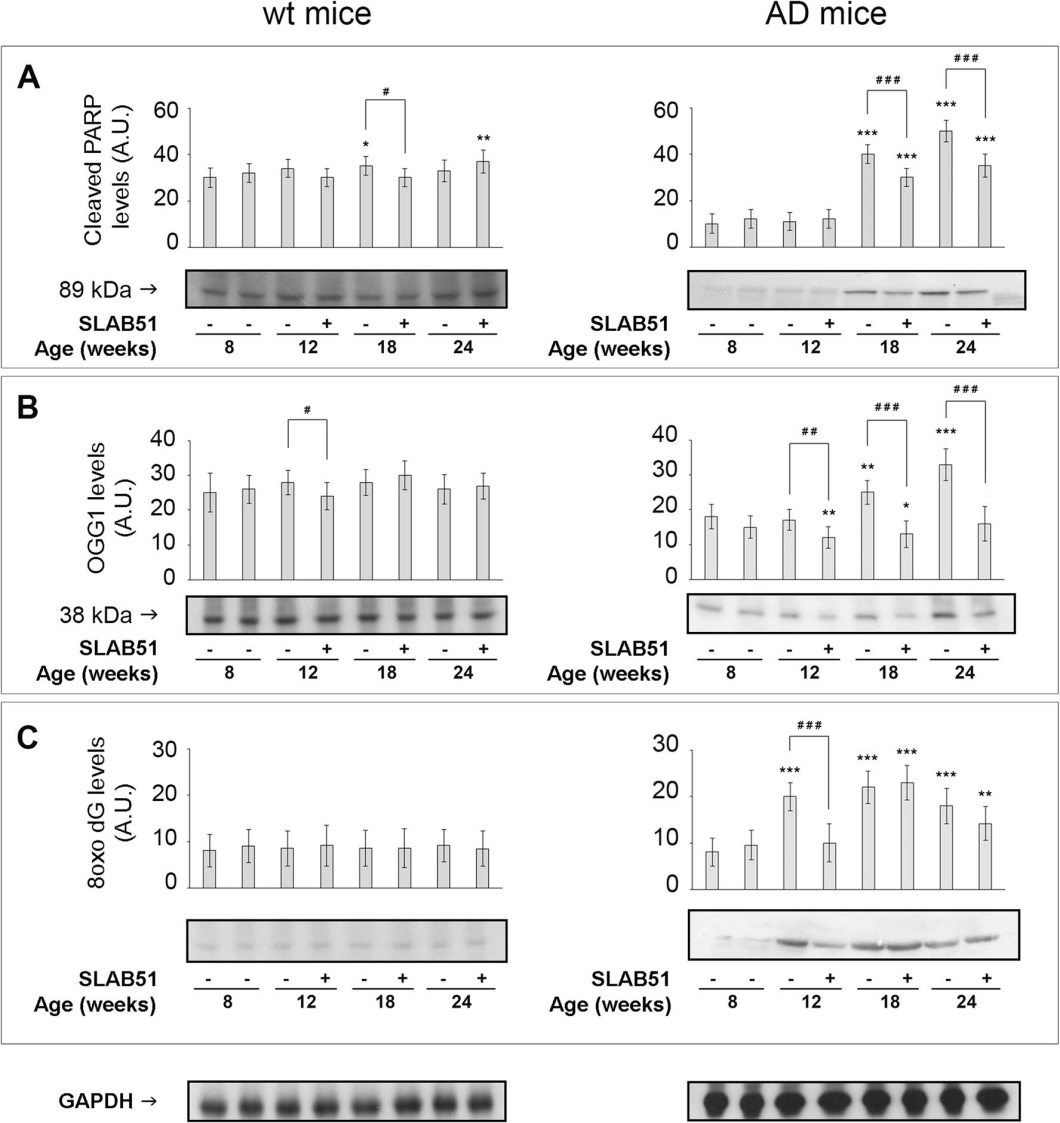
Effect of SLAB51 on DNA oxidation and repair mechanisms. Cleaved PARP (**a**), OGG1 (**b**) and 8-oxodG (**c**) levels measured in brain homogenates of SLAB51-treated and SLAB51-untreated wt (left) and AD (right) mice. The densitometric analyses obtained from five separate blots and representative immunoblots are shown. Equal protein loading was verified by using an anti-GAPDH antibody. The detection was performed with an ECL Western blotting analysis system. Statistical significance compared to untreated 8-week-old mice and age-matched mice is indicated with asterisks (*p < 0.05; **p < 0.01; ***p < 0.001) and hashtags (^#^*p* < 0.05; ^##^*p* < 0.01; ^###^*p* < 0.001), respectively

Minor changes were observed in wt mice in the markers of DNA oxidative damage with time (PARP, 18-week-old mice) and treatment (OGG1, 12-week-old mice; PARP, 24 weeks old) (Fig. 6, left insets). PARP and OGG1 are differently involved in DNA repair mechanisms: PARP mediates DNA breaks repair, whereas OGG1 removes 8-oxoguanine base lesions generated by ROS. Interestingly, a direct binding between OGG1 and PARP1, enhanced by oxidative stress, was recently evidenced [41]. In untreated AD mice, cleaved PARP levels increased at 18 and 24 weeks of age, and SLAB51 treatment only partially (but significantly) restored initial conditions (Fig. 6a).

Similarly, OGG1 levels increased at 18 and 24 weeks of age in untreated AD mice, but in this case SLAB51 was extremely effective in restoring basal levels of 8-week-old untreated AD mice (at 12 weeks, SLAB51 decreased OGG1 levels below basal levels of 8-week-old untreated AD mice) (Fig. 6b).

8-oxodg levels reached the maximum increase yet at 12 weeks of age in untreated AD mice, and SLAB51 treatment successfully restored basal levels of 8-week-old untreated AD mice only at the earliest time point (Fig. 6c).

## Discussion

Growing evidence supports the use of probiotics for the rational manipulation of gut microbiota by virtue of their positive influence on CNS-associated disorders and functions [25, 27, 42]. In fact, a strict relationship/communication exists between the gastrointestinal tract and the brain, the so-called gut-brain axis, which is mediated by different pathways that include hormonal, neural and immune stimuli [43].

We have recently shown that the modulation of gut microbiota by using SLAB51 formulation (a mixture of lactic acid bacteria and bifidobacteria) affects numerous neuronal pathways, with a significant delay of AD progression in 3xTg-AD mice [28]. In details, SLAB51 changed microbiota composition and metabolites, favouring the proliferation of anti-inflammatory species and positively interfering with the concentration of gut hormones and peptides extremely important in regulating energy homeostasis and food intake, and was able to affect the CNS modulating nervous functions [44–49]. The downstream effects of these changes included the modulation of neuronal proteolysis, the reduction of Aβ load and the improvement of cognitive functions, suggesting a role for probiotics in the prevention of AD and their possible application in AD preventative therapy [28].

The present study was conceived to explore the ability of SLAB51 to counteract oxidative stress, a condition that is exacerbated in the brain of AD subjects, in 3xTg-AD mice, investigating the molecular mechanisms involved. We first focused the attention on the role of SIRT1, a deacetylase with a strong neuroprotective and antioxidant potential that regulates the expression of several antioxidant genes, including SOD, CAT and peroxiredoxins 3 and 5 [21, 50–57]. In the brain of untreated AD mice, we observed a great, age-dependent loss of SIRT1 functionality and expression levels that negatively mediates a series of processes related to cell survival and metabolism. In fact, the deleterious effects of decreased SIRT1 expression are widely recognized, including the accumulation of Aβ and tau in the cerebral cortex of patients with AD [58]. Our data demonstrate that both SIRT1 activity and expression were significantly increased in the brain of AD mice administered with the probiotic formulation SLAB51. The activation of SIRT1 was also confirmed by the data on the acetylation levels of its substrate RARβ. In fact, if an age-dependent increase in the degree of acetylation was detected in untreated AD mice, in line with the diminished expression of SIRT1, SLAB51 strongly reduced the amount of RARβ acetylated lysines by restoring SIRT1 levels. Interestingly, the deacetylation and the consequent activation of RARβ stimulate ADAM10 gene transcription stimulating the non-amyloidogenic pathway of APP processing and preventing Aβ peptide generation and deposition [23, 59]. These data are in accordance with our previous studies reporting diminished deposition of Aβ toxic fragments in the brain of AD mice treated with SLAB51 [28].

SIRT1 activity also regulates p53 acetylation [60, 61]. p53 is stabilized upon acetylation, thus enhancing its apoptotic activity. It has been shown that the activation of SIRT1 by resveratrol reduced acetylation of p53 at lysine 382 and downregulated p53 [20]. Additionally, human SIRT1 can directly bind p53 both in vitro and in vivo and promotes cell survival under stress by specifically repressing p53-dependent apoptotic response [62]. In AD mice treated with the probiotic mixture, increased levels of SIRT1 inversely correlated with acetylated p53, suggesting the protective action of SLAB51 against p53-mediated apoptosis. In line with previously published data on the level of p53 in AD brain, we observed higher level of the protein in the brain of untreated AD mice [63, 64]. These elevated p53 levels were proposed to favour tau phosphorylation in HEK293a cells [63]. p53 behaviour is in agreement with our previous findings on UPS functionality; being p53 is a well-known proteasome substrate, it is reasonable that it accumulates in AD mice with impaired UPS-mediated proteolysis, and that SLAB51 administration results in decreased levels of proteasome substrates (among these p53), due to restored proteasome activities [28]. Moreover, p53 deacetylation by activated SIRT1 is also related to ghrelin increased plasma levels in the same AD mouse model upon SLAB51 oral administration, as we have previously shown [28]. In fact, ghrelin has been demonstrated to promote the hypothalamic SIRT1-p53 pathway, causing changes in fatty acids metabolism and feeding behaviour [65]. These data are in agreement with our previous work reporting an enriched gut concentration of anti-inflammatory short chain fatty acids and a decrease of pro-inflammatory cytokine levels in SLAB51-treated AD mice [28]. Collectively, these data strongly support the idea that SLAB51 activates SIRT1 pathway, this latter representing a link between metabolism and inflammation [66]. In fact, inhibition of SIRT1 activity by oxidative stress reduces its levels by posttranslational modifications, favouring its nucleocytoplasmic shuttling and determining the accumulation of transcription factors and modifications of histones H3 and H4. These events finally cause the abnormal transcription of pro-inflammatory, prosenescent and proapoptotic mediators [67].

Being SIRT1 directly involved in the regulation of the oxidative stress, whose levels were demonstrated to be extremely high in the brain of AD subjects [68, 69], we measured the functionality of redox enzymes and the amount of well-established markers of proteins, lipid and DNA oxidation to evaluate if the SLAB51-induced activation of SIRT1 pathway effectively corresponded to an antioxidant action. We observed severe age-dependent alterations of the oxidative status in the brains of AD transgenic mice. Compared to the 8-week-old animals, elder AD individuals showed decreased activities of antioxidant enzymes, mainly GPx and catalase, despite of the increased expression levels of such enzymes. These data are in agreement with other authors’ evidence that describes a “redistribution phenomenon,” with the enzymes being more concentrated in the oxidized sites but dramatically impaired in their functionality [70]. Simultaneously, an intense enhancement of macromolecule oxidation markers, including carbonyls, 3-NT, 4-HNE and 8-oxodG, was detected in AD brains. Interestingly, in AD mice SLAB51 administration markedly mitigated oxidative stress-related damages as indicated by the evident increase in the activity of antioxidant enzymes and the diminished levels of macromolecule oxidation markers.

Finally, PARP cleavage and OGG1 levels increased in AD-untreated mice in response to the accumulation of oxidized species of DNA [71]. This result is in agreement with the suppression of SIRT1 expression in the same mice. In fact, PARP and SIRT1 share NAD^+^ as cofactor [72], thus PARP1 activation can inhibit SIRT1 functionality [73].

Collectively, these data demonstrated the great impact of SIRT1 pathway reactivation upon SLAB51 administration in preserving brain redox homeostasis, with positive outcomes for AD. This property makes SLAB51 able to act at different levels in the cell, by exerting beneficial effects that definitely ameliorate the symptomatology of AD. These findings further contribute to unveil the innovative role for microbiota manipulative strategies for future AD (co)therapies involving probiotic approaches.

## Electronic Supplementary Material

ESM 1 (DOCX 783 kb).

## References

[1] Bloom GS (2014). Amyloid-beta and tau: the trigger and bullet in Alzheimer disease pathogenesis. JAMA neurology.

[2] Varadarajan S, Yatin S, Aksenova M, Butterfield DA (2000). Review: Alzheimer’s amyloid beta-peptide-associated free radical oxidative stress and neurotoxicity. J Struct Biol.

[3] Halliwell B (2006). Oxidative stress and neurodegeneration: where are we now?. J Neurochem.

[4] Kim GH, Kim JE, Rhie SJ, Yoon S (2015). The role of oxidative stress in neurodegenerative diseases. Exp Neurobiol.

[5] Sultana R, Perluigi M, Butterfield DA (2009). Oxidatively modified proteins in Alzheimer’s disease (AD), mild cognitive impairment and animal models of AD: role of Abeta in pathogenesis. Acta Neuropathol.

[6] Dawnay AB, Millar DJ (1997). Glycation and advanced glycation end-product formation with icodextrin and dextrose. Perit Dial Int: J Int Soc Perit Dial.

[7] Di Domenico F, Pupo G, Giraldo E, Badia MC, Monllor P, Lloret A, Eugenia Schinina M, Giorgi A, Cini C, Tramutola A, Butterfield DA, Vina J, Perluigi M (2016). Oxidative signature of cerebrospinal fluid from mild cognitive impairment and Alzheimer disease patients. Free Radic Biol Med.

[8] Shen L, Chen C, Yang A, Chen Y, Liu Q, Ni J (2015). Redox proteomics identification of specifically carbonylated proteins in the hippocampi of triple transgenic Alzheimer’s disease mice at its earliest pathological stage. J Proteome.

[9] Butterfield DA, Reed T, Perluigi M, De Marco C, Coccia R, Cini C, Sultana R (2006). Elevated protein-bound levels of the lipid peroxidation product, 4-hydroxy-2-nonenal, in brain from persons with mild cognitive impairment. Neurosci Lett.

[10] Butterfield DA, Bader Lange ML, Sultana R (2010). Involvements of the lipid peroxidation product, HNE, in the pathogenesis and progression of Alzheimer’s disease. Biochim Biophys Acta.

[11] Arimon M, Takeda S, Post KL, Svirsky S, Hyman BT, Berezovska O (2015). Oxidative stress and lipid peroxidation are upstream of amyloid pathology. Neurobiol Dis.

[12] Cooke MS, Evans MD, Dizdaroglu M, Lunec J (2003). Oxidative DNA damage: mechanisms, mutation, and disease. FASEB J: Off Publ Fed Am Soc Exp Biol.

[13] Gabbita SP, Lovell MA, Markesbery WR (1998). Increased nuclear DNA oxidation in the brain in Alzheimer’s disease. J Neurochem.

[14] Wang J, Xiong S, Xie C, Markesbery WR, Lovell MA (2005). Increased oxidative damage in nuclear and mitochondrial DNA in Alzheimer’s disease. J Neurochem.

[15] Ding Q, Markesbery WR, Cecarini V, Keller JN (2006). Decreased RNA, and increased RNA oxidation, in ribosomes from early Alzheimer’s disease. Neurochem Res.

[16] Marcus DL, Thomas C, Rodriguez C, Simberkoff K, Tsai JS, Strafaci JA, Freedman ML (1998). Increased peroxidation and reduced antioxidant enzyme activity in Alzheimer’s disease. Exp Neurol.

[17] Pocernich CB, Butterfield DA (2012). Elevation of glutathione as a therapeutic strategy in Alzheimer disease. Biochim Biophys Acta.

[18] Shao C, Xiong S, Li GM, Gu L, Mao G, Markesbery WR, Lovell MA (2008). Altered 8-oxoguanine glycosylase in mild cognitive impairment and late-stage Alzheimer’s disease brain. Free Radic Biol Med.

[19] Horio Y, Hayashi T, Kuno A, Kunimoto R (2011). Cellular and molecular effects of sirtuins in health and disease. Clin Sci.

[20] Kim D, Nguyen MD, Dobbin MM, Fischer A, Sananbenesi F, Rodgers JT, Delalle I, Baur JA, Sui G, Armour SM, Puigserver P, Sinclair DA, Tsai LH (2007). SIRT1 deacetylase protects against neurodegeneration in models for Alzheimer’s disease and amyotrophic lateral sclerosis. EMBO J.

[21] Hori YS, Kuno A, Hosoda R, Horio Y (2013). Regulation of FOXOs and p53 by SIRT1 modulators under oxidative stress. PLoS One.

[22] Solomon JM, Pasupuleti R, Xu L, McDonagh T, Curtis R, DiStefano PS, Huber LJ (2006). Inhibition of SIRT1 catalytic activity increases p53 acetylation but does not alter cell survival following DNA damage. Mol Cell Biol.

[23] Lee HR, Shin HK, Park SY, Kim HY, Lee WS, Rhim BY, Hong KW, Kim CD (2014). Cilostazol suppresses beta-amyloid production by activating a disintegrin and metalloproteinase 10 via the upregulation of SIRT1-coupled retinoic acid receptor-beta. J Neurosci Res.

[24] Wang Y, Kasper LH (2014). The role of microbiome in central nervous system disorders. Brain Behav Immun.

[25] Ghaisas S, Maher J, Kanthasamy A (2016). Gut microbiome in health and disease: Linking the microbiome-gut-brain axis and environmental factors in the pathogenesis of systemic and neurodegenerative diseases. Pharmacol Ther.

[26] Bhattacharjee S, Lukiw WJ (2013). Alzheimer’s disease and the microbiome. Front Cell Neurosci.

[27] Distrutti E, O’Reilly JA, McDonald C, Cipriani S, Renga B, Lynch MA, Fiorucci S (2014). Modulation of intestinal microbiota by the probiotic VSL#3 resets brain gene expression and ameliorates the age-related deficit in LTP. PLoS One.

[28] Bonfili L, Cecarini V, Berardi S, Scarpona S, Suchodolski JS, Nasuti C, Fiorini D, Boarelli MC, Rossi G, Eleuteri AM (2017). Microbiota modulation counteracts Alzheimer’s disease progression influencing neuronal proteolysis and gut hormones plasma levels. Sci Rep.

[29] Oddo S, Caccamo A, Shepherd JD, Murphy MP, Golde TE, Kayed R, Metherate R, Mattson MP, Akbari Y, LaFerla FM (2003). Triple-transgenic model of Alzheimer’s disease with plaques and tangles: intracellular Abeta and synaptic dysfunction. Neuron.

[30] Crawford JD, Terry ME, Rourke GM (1950). Simplification of drug dosage calculation by application of the surface area principle. Pediatrics.

[31] Bradford MM (1976). A rapid and sensitive method for the quantitation of microgram quantities of protein utilizing the principle of protein-dye binding. Anal Biochem.

[32] Wegener D, Hildmann C, Riester D, Schwienhorst A (2003). Improved fluorogenic histone deacetylase assay for high-throughput-screening applications. Anal Biochem.

[33] Aebi H (1984). Catalase in vitro. Methods Enzymol.

[34] Habig WH, Pabst MJ, Jakoby WB (1974). Glutathione S-transferases: the first enzymatic step in mercapturic acid formation. J Biol Chem.

[35] Mannervik B, Danielson UH (1988). Glutathione transferases—structure and catalytic activity. CRC Crit Rev Biochem.

[36] Wilce MC, Parker MW (1994). Structure and function of glutathione S-transferases. Biochim Biophys Acta.

[37] Gupta BL, Baquer NZ (1998). Hexokinase, glucose-6-phosphate dehydrogenase and antioxidant enzymes in diabetic reticulocytes: effects of insulin and vanadate. Biochem Mol Biol Int.

[38] Mannervik B (1985). Glutathione peroxidase. Methods Enzymol.

[39] Thorpe GH, Kricka LJ, Moseley SB, Whitehead TP (1985). Phenols as enhancers of the chemiluminescent horseradish peroxidase-luminol-hydrogen peroxide reaction: application in luminescence-monitored enzyme immunoassays. Clin Chem.

[40] Reed SM, Quelle DE (2014). p53 acetylation: regulation and consequences. Cancers.

[41] Noren Hooten N, Kompaniez K, Barnes J, Lohani A, Evans MK (2011). Poly(ADP-ribose) polymerase 1 (PARP-1) binds to 8-oxoguanine-DNA glycosylase (OGG1). J Biol Chem.

[42] Felice VD, Quigley EM, Sullivan AM, OKeeffe GW, O’Mahony SM (2016). Microbiota-gut-brain signalling in Parkinson’s disease: implications for non-motor symptoms. Parkinsonism Relat Disord.

[43] Cryan JF, O’Mahony SM (2011). The microbiome-gut-brain axis: from bowel to behavior. Neurogastroenterol Motil: Off J Eur Gastrointest Motil Soc.

[44] Shi L, Du X, Jiang H, Xie J (2017). Ghrelin and neurodegenerative disorders-a review. Mol Neurobiol.

[45] Signore AP, Zhang F, Weng Z, Gao Y, Chen J (2008). Leptin neuroprotection in the CNS: mechanisms and therapeutic potentials. J Neurochem.

[46] Gomes S, Martins I, Fonseca AC, Oliveira CR, Resende R, Pereira CM (2014). Protective effect of leptin and ghrelin against toxicity induced by amyloid-beta oligomers in a hypothalamic cell line. J Neuroendocrinol.

[47] Stoyanova II (2014) Ghrelin: a link between ageing, metabolism and neurodegenerative disorders. Neurobiol Dis:72–72 Pt A, 83. 10.1016/j.nbd.2014.08.026 10.1016/j.nbd.2014.08.026 25173805

[48] Folch J, Patraca I, Martinez N, Pedros I, Petrov D, Ettcheto M, Abad S, Marin M, Beas-Zarate C, Camins A (2015). The role of leptin in the sporadic form of Alzheimer’s disease. Interactions with the adipokines amylin, ghrelin and the pituitary hormone prolactin. Life Sci.

[49] Theodoropoulou A, Metallinos IC, Psyrogiannis A, Vagenakis GA, Kyriazopoulou V (2012). Ghrelin and leptin secretion in patients with moderate Alzheimer’s disease. J Nutr Health Aging.

[50] Paraiso AF, Mendes KL, Santos SH (2013). Brain activation of SIRT1: role in neuropathology. Mol Neurobiol.

[51] Lou Y, Wang Z, Xu Y, Zhou P, Cao J, Li Y, Chen Y, Sun J, Fu L (2015). Resveratrol prevents doxorubicin-induced cardiotoxicity in H9c2 cells through the inhibition of endoplasmic reticulum stress and the activation of the Sirt1 pathway. Int J Mol Med.

[52] Liu Z, Jiang C, Zhang J, Liu B, Du Q (2016). Resveratrol inhibits inflammation and ameliorates insulin resistant endothelial dysfunction via regulation of AMP-activated protein kinase and sirtuin 1 activities. J Diabetes.

[53] Olmos Y, Sanchez-Gomez FJ, Wild B, Garcia-Quintans N, Cabezudo S, Lamas S, Monsalve M (2013). SirT1 regulation of antioxidant genes is dependent on the formation of a FoxO3a/PGC-1alpha complex. Antioxid Redox Signal.

[54] Tanno M, Kuno A, Yano T, Miura T, Hisahara S, Ishikawa S, Shimamoto K, Horio Y (2010). Induction of manganese superoxide dismutase by nuclear translocation and activation of SIRT1 promotes cell survival in chronic heart failure. J Biol Chem.

[55] Hasegawa K, Wakino S, Yoshioka K, Tatematsu S, Hara Y, Minakuchi H, Washida N, Tokuyama H, Hayashi K, Itoh H (2008). Sirt1 protects against oxidative stress-induced renal tubular cell apoptosis by the bidirectional regulation of catalase expression. Biochem Biophys Res Commun.

[56] Salminen A, Kaarniranta K, Kauppinen A (2013). Crosstalk between oxidative stress and SIRT1: impact on the aging process. Int J Mol Sci.

[57] Brunet A, Sweeney LB, Sturgill JF, Chua KF, Greer PL, Lin Y, Tran H, Ross SE, Mostoslavsky R, Cohen HY, Hu LS, Cheng HL, Jedrychowski MP, Gygi SP, Sinclair DA, Alt FW, Greenberg ME (2004). Stress-dependent regulation of FOXO transcription factors by the SIRT1 deacetylase. Science.

[58] Julien C, Tremblay C, Émond V, Lebbadi M, Norman Salem DAB, Calon F (2009). SIRT1 decrease parallels the accumulation of tau in Alzheimer disease. J Neuropathol Exp Neurol.

[59] Qin W, Yang T, Ho L, Zhao Z, Wang J, Chen L, Zhao W, Thiyagarajan M, MacGrogan D, Rodgers JT, Puigserver P, Sadoshima J, Deng H, Pedrini S, Gandy S, Sauve AA, Pasinetti GM (2006). Neuronal SIRT1 activation as a novel mechanism underlying the prevention of Alzheimer disease amyloid neuropathology by calorie restriction. J Biol Chem.

[60] Vaziri H, Dessain SK, Ng Eaton E, Imai SI, Frye RA, Pandita TK, Guarente L, Weinberg RA (2001). hSIR2(SIRT1) functions as an NAD-dependent p53 deacetylase. Cell.

[61] Zhang C, Feng Y, Qu S, Wei X, Zhu H, Luo Q, Liu M, Chen G, Xiao X (2011). Resveratrol attenuates doxorubicin-induced cardiomyocyte apoptosis in mice through SIRT1-mediated deacetylation of p53. Cardiovasc Res.

[62] Luo J, Nikolaev AY, Imai S, Chen D, Su F, Shiloh A, Guarente L, Gu W (2001). Negative control of p53 by Sir2alpha promotes cell survival under stress. Cell.

[63] Hooper C, Meimaridou E, Tavassoli M, Melino G, Lovestone S, Killick R (2007). p53 is upregulated in Alzheimer’s disease and induces tau phosphorylation in HEK293a cells. Neurosci Lett.

[64] Kitamura Y, Shimohama S, Kamoshima W, Matsuoka Y, Nomura Y, Taniguchi T (1997). Changes of p53 in the brains of patients with Alzheimer’s disease. Biochem Biophys Res Commun.

[65] Velasquez DA, Martinez G, Romero A, Vazquez MJ, Boit KD, Dopeso-Reyes IG, Lopez M, Vidal A, Nogueiras R, Dieguez C (2011). The central sirtuin 1/p53 pathway is essential for the orexigenic action of ghrelin. Diabetes.

[66] Vachharajani VT, Liu T, Wang X, Hoth JJ, Yoza BK, McCall CE (2016). Sirtuins link inflammation and metabolism. J Immunol Res.

[67] Hwang JW, Yao H, Caito S, Sundar IK, Rahman I (2013). Redox regulation of SIRT1 in inflammation and cellular senescence. Free Radic Biol Med.

[68] Perry G, Cash AD, Smith MA (2002). Alzheimer disease and oxidative stress. J Biomed Biotechnol.

[69] Zhao Y, Zhao B (2013). Oxidative stress and the pathogenesis of Alzheimer’s disease. Oxidative Med Cell Longev.

[70] Omar RA, Chyan YJ, Andorn AC, Poeggeler B, Robakis NK, Pappolla MA (1999). Increased expression but reduced activity of antioxidant enzymes in Alzheimer’s disease. J Alzheimer’s Dis: JAD.

[71] Martire S, Mosca L, d’Erme M (2015). PARP-1 involvement in neurodegeneration: a focus on Alzheimer’s and Parkinson’s diseases. Mech Ageing Dev.

[72] Canto C, Auwerx J (2011). Interference between PARPs and SIRT1: a novel approach to healthy ageing?. Aging.

[73] Bai P, Canto C, Oudart H, Brunyanszki A, Cen Y, Thomas C, Yamamoto H, Huber A, Kiss B, Houtkooper RH, Schoonjans K, Schreiber V, Sauve AA, Menissier-de Murcia J, Auwerx J (2011). PARP-1 inhibition increases mitochondrial metabolism through SIRT1 activation. Cell Metab.

