# Supplementary material for: SLAB51 Probiotic Formulation Activates SIRT1 Pathway Promoting Antioxidant and Neuroprotective Effects in an AD Mouse Model
Source: Mol Neurobiol. 2018 Feb 28;55(10):7987–8000. doi: 10.1007/s12035-018-0973-4 (PMC6132798; doi:10.1007/s12035-018-0973-4)
Supplement: Supplementary file 1 — (DOCX 783 kb). [file 12035_2018_973_MOESM1_ESM.docx]

**Supplementary figures**


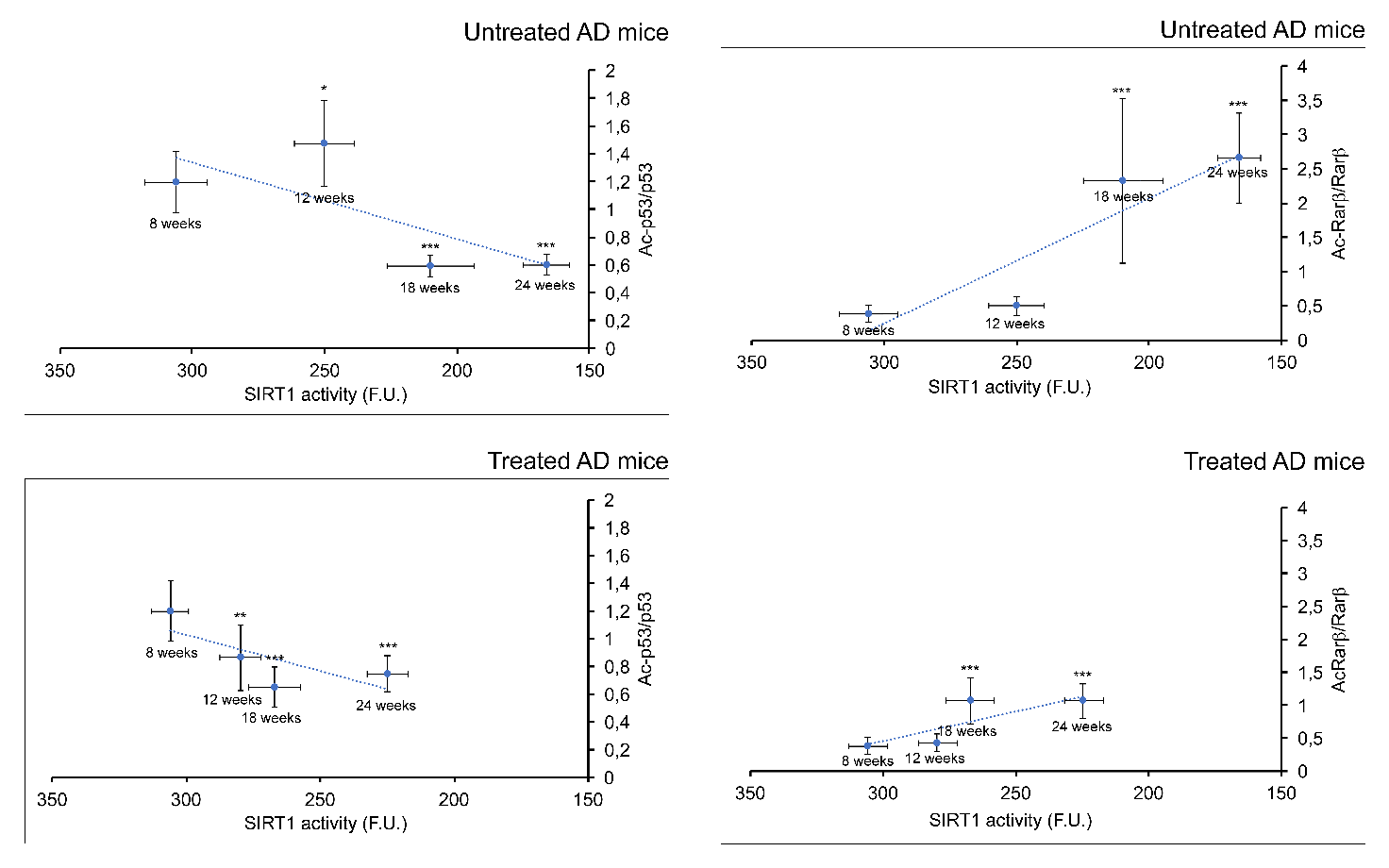


**Supp. Fig.1:** Scatter-plots of the dependence of Ac-p53/p53 and Ac-RARβ/RARβ ratios from SIRT1 activity/time in treated and untreated AD mice. Statistical significance compared to corresponding 8-week-old mice is indicated with § mark (^§^p< 0.05; ^§§^p< 0.01; ^§§§^p< 0.001).


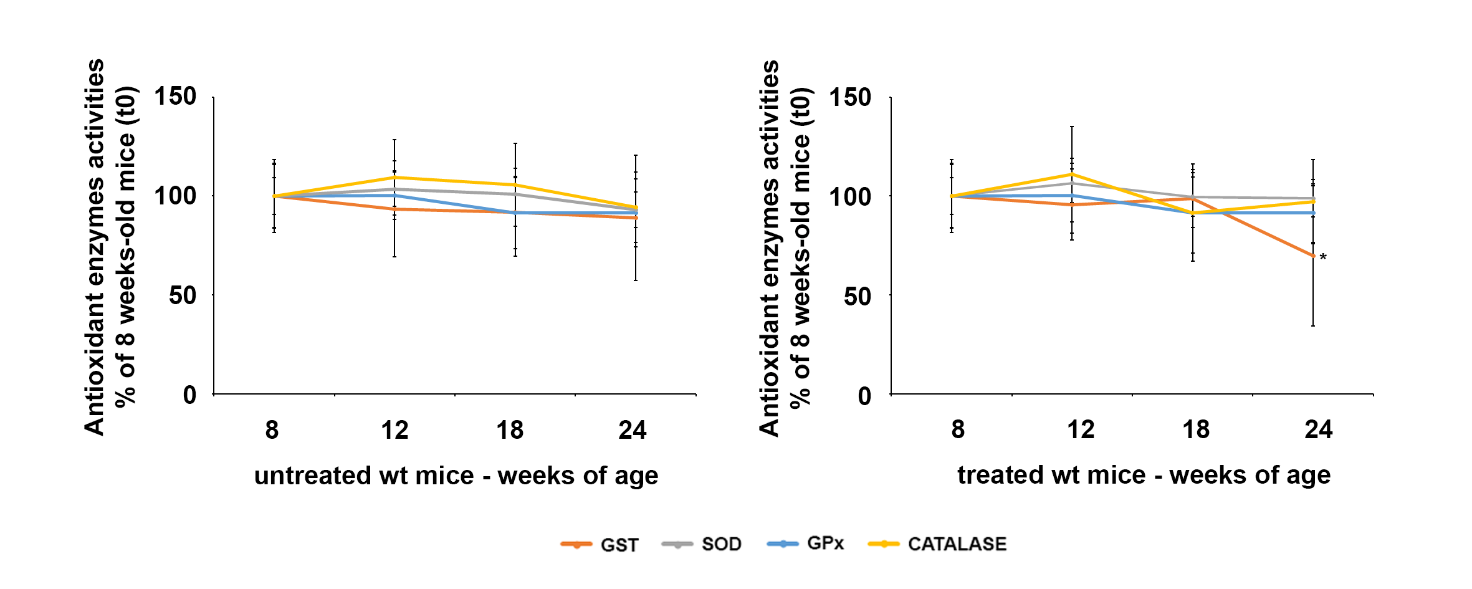


**Supp. Fig.2:** Changes in GST, GPx, SOD and catalase activities in the brain of ageing untreated and treated wt mice. Enzyme activities are presented as percentage of 8-week-old mice. Statistical significance compared to corresponding 8-week-old mice is indicated with asterisks (*p< 0.05; **p< 0.01; ***p< 0.001).

**
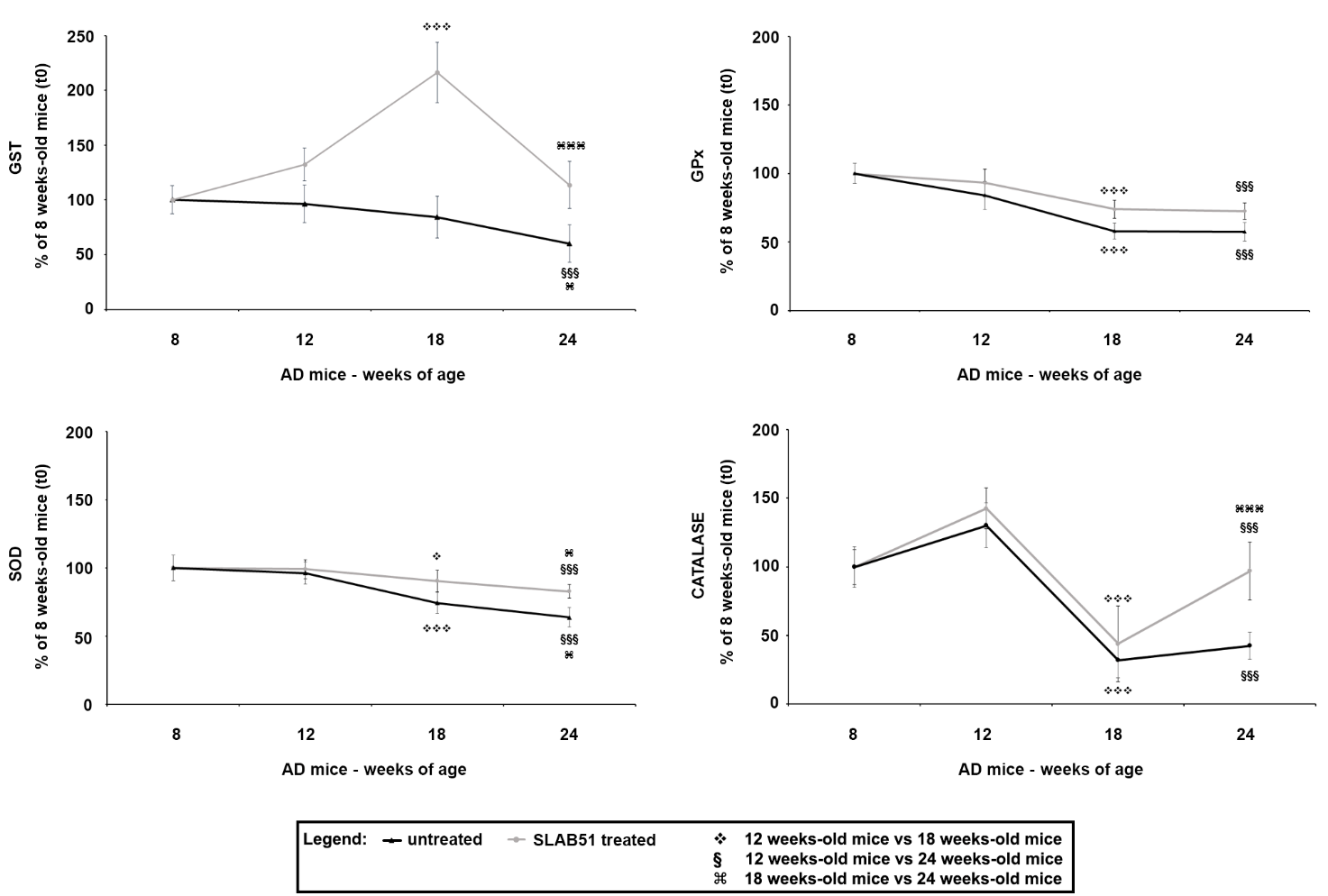
**

**Supp. Fig.3:** Comparison of redox enzymes activities in the brain of treated and untreated AD mice


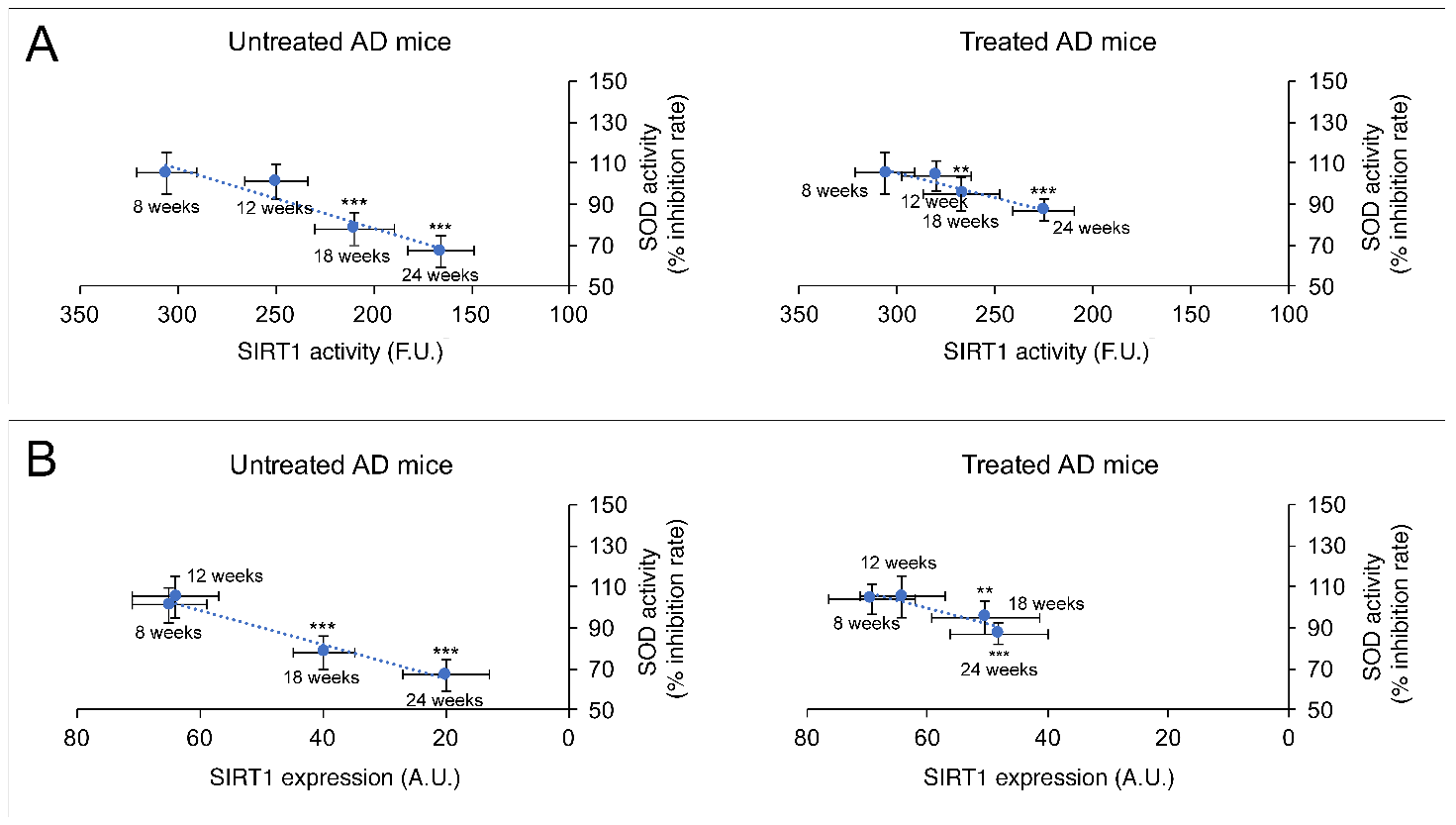


**Supp. Fig.4:** Scatter-plots of the dependence of SOD activity from SIRT1 activity/expression in treated and untreated AD mice. Statistical significance compared to corresponding 8-week-old mice is indicated with asterisks (*p< 0.05; **p< 0.01; ***p< 0.001).


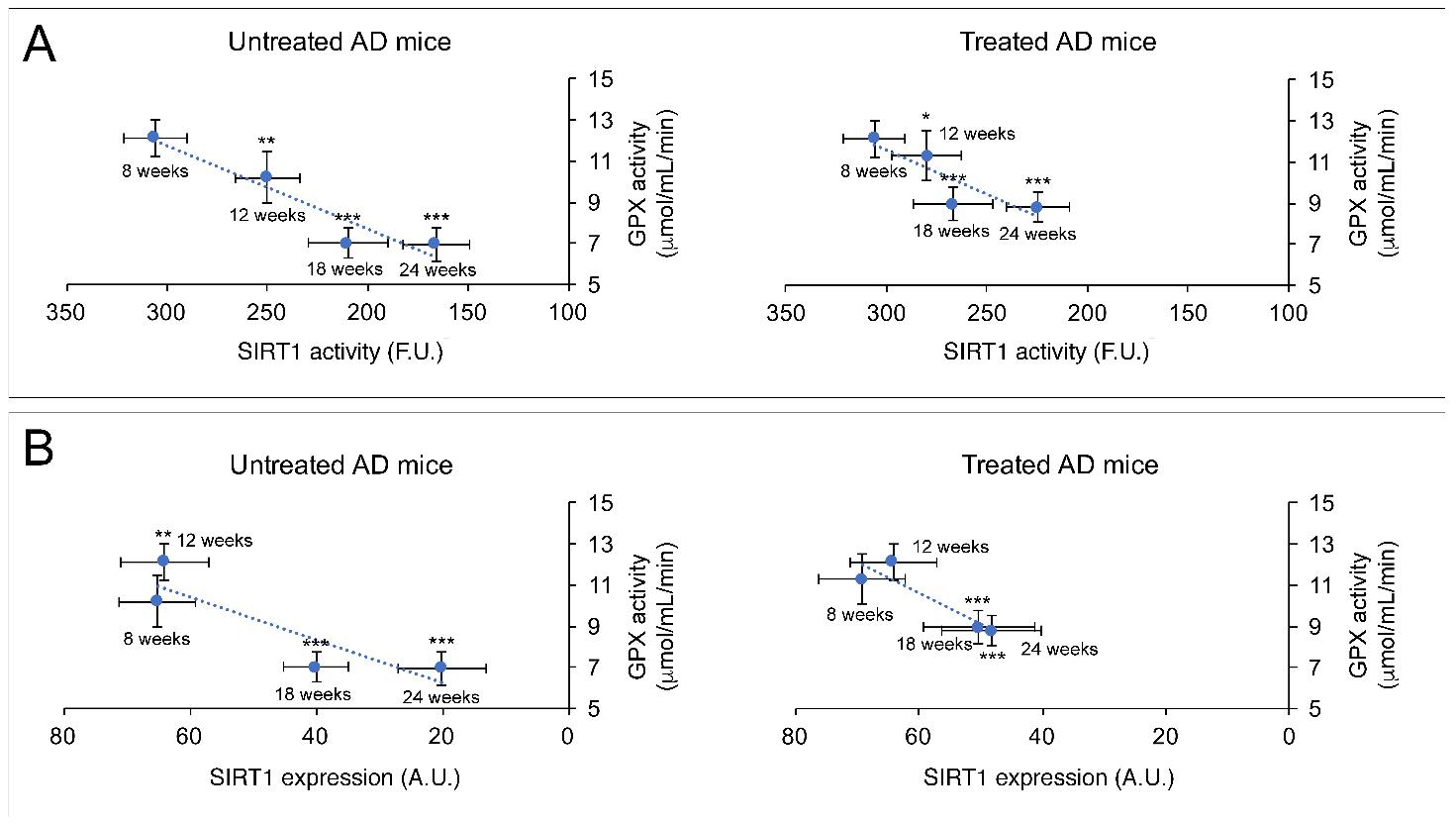


**Supp. Fig.5:** Scatter-plots of the dependence of GPx activity from SIRT1 activity/expression in treated and untreated AD mice. Statistical significance compared to corresponding 8-week-old mice is indicated with asterisks (*p< 0.05; **p< 0.01; ***p< 0.001).
